# Brief intermittent intense exercise as interoceptive exposure for panic disorder: a randomized controlled clinical trial

**DOI:** 10.3389/fpsyt.2025.1739639

**Published:** 2026-02-09

**Authors:** Ricardo William Muotri, Alan Campos Luciano, Alia Garrudo Guirado, Francisco Lotufo Neto, Márcio Bernik

**Affiliations:** 1 Anxiety Disorders Program, Institute of Psychiatry, University of Sao Paulo (USP), Sao Paulo, Brazil; 2 Institute of Mathematics and Statistics, University of Sao Paulo (USP), Sao Paulo, Brazil

**Keywords:** panic disorder, anxiety disorders, interoception, exercise, exercise therapy, behavior therapy

## Abstract

**Background:**

Interoceptive exposure (IE) to feared bodily sensations is a core component of cognitive–behavioral therapy for panic disorder (PD), but standard office-based IE can be perceived as aversive and tedious, potentially limiting engagement. Vigorous physical exercise may provide a more acceptable and health-promoting way to elicit interoceptive cues. Objective: To examine the feasibility and efficacy of a brief intermittent intense exercise (BIE) program, used as an IE strategy, compared with Jacobson’s relaxation training (RT) in treatment-free patients with PD.

**Methods:**

In this prospective, parallel-group, randomized, assessor-blinded clinical trial, 72 sedentary adults with PD (34 men; mean age 33.3 ± 7.7 years), free of pharmacological treatment for ≥12 weeks, were allocated to either a 12-week BIE program (n = 37) or RT (n = 35). BIE consisted of supervised walking interspersed with repeated 30-s high-intensity sprints, while RT followed a standardized progressive muscular relaxation protocol. All participants received identical placebo medication. The primary outcome was Panic Agoraphobia Scale (PAS) score, assessed by a blinded rater at baseline and weeks 6, 12, and 24 (follow-up). Secondary outcomes included frequency and intensity of panic attacks, Hamilton Anxiety Rating Scale (HAM-A), and Hamilton Depression Rating Scale (HAM-D) scores.

**Results:**

Both groups improved over time, but a significant group × time interaction favored BIE on PAS scores (F = 56.1, p < 0.001, η² = 0.46). At week 12, PAS scores were lower in the BIE group than in RT (14.9 ± 5.3 vs. 23.1 ± 9.4; t = −4.72, p < 0.001), and this difference was maintained at week 24 (14.2 ± 5.5 vs. 24.7 ± 8.5; t = −6.07, p < 0.001). At follow-up, BIE also yielded fewer panic attacks (0.7 ± 0.6 vs. 1.5 ± 1.0; t = 3.79, p = 0.003) and lower HAM-D scores (13.3 ± 4.7 vs. 16.4 ± 5.6; t = −2.55, p = 0.013).

**Conclusion:**

A 12-week BIE program used as interoceptive exposure was feasible and more effective than relaxation training in reducing panic symptom severity and panic attack frequency, with effects sustained for at least 24 weeks. These findings support the incorporation of structured exercise-based IE into PD treatment programs as a low-cost and engaging option.

**Clinial trial registration:**

https://www.clinicaltrials.gov, identifier NCT06073691.

## Highlights

What are the new findings?

- A 12-week program of brief intermittent intense exercise (BIE), used as interoceptive exposure, was feasible and more effective than relaxation training in reducing panic disorder severity at endpoint and at 24-week follow-up.
- Patients undergoing BIE experienced fewer and less severe panic attacks at follow-up compared with those receiving relaxation training.

How might this impact clinical practice in the future?

- Structured exercise protocols such as BIE can be integrated into treatment programs for panic disorder as an interoceptive exposure strategy.
- This treatment option is low cost, health-promoting, and can enhance patient engagement by providing an interoceptive exposure program that is experienced as more enjoyable than traditional office-based procedures.

## Introduction

Panic attacks (PA) are characterized by abrupt surges of intense fear accompanied by marked autonomic arousal. Over time, these physiological symptoms of arousal tend to be perceived as dangerous in patients with panic disorder (PD), leading to a heightened awareness of somatic sensations and catastrophic misinterpretations of bodily cues (1–3). Increased attention to internal sensations has been documented in PD (4, 5), and anxiety and panic can even be induced experimentally using false heart rate feedback in these patients (6). A common consequence is the avoidance of situations and activities associated with physical effort, which often results in sedentary behavior (7).

Stampler proposed an integrated model in which autonomic hyperactivity becomes an interoceptive conditioned stimulus that elicits further anxiety and threat perception (8). This model is supported by experimental work demonstrating that defensive reactivity in PD ranges from anxious apprehension to full-blown panic as interoceptive threat proximity increases (9). The tendency to overestimate and fear anxiety-related bodily sensations and their consequences is commonly referred to as anxiety sensitivity (3, 10), and is particularly relevant in PD. In parallel, PD patients may show impaired interoceptive accuracy, as evidenced by difficulties in judging exertion during ergospirometry tests and in using perceived exertion to identify the anaerobic threshold (2, 11).

Cognitive-behavioral therapy (CBT) for PD typically combines several evidence-based components (12–14). Among these, interoceptive exposure (IE) has been identified as a core ingredient in effective treatment protocols. IE-inclusive CBT programs have shown superior outcomes on panic frequency, global severity, and functional impairment compared with protocols that omit IE. For example, Craske et al. (15) reported that a CBT intervention combining IE, cognitive restructuring, and *in vivo* exposure produced robust and durable improvements, whereas IE was more effective than breathing retraining in reducing panic frequency, phobic fears, and general anxiety.

IE protocols repeatedly elicit feared bodily sensations associated with PA (e.g., dyspnea, palpitations, dizziness) to increase tolerance and reduce distress (16). Conventional IE is usually delivered through office-based exercises, such as voluntary hyperventilation or spinning on a chair (17, 18), and such a protocol has been used for many years in our clinic for patients with PD (19). Although these procedures are effective, a substantial proportion of patients either do not respond adequately or drop out of treatment (21, 22). One possible explanation is that office-based IE can be perceived as artificial and highly aversive (23).

Vigorous physical activity may represent a more natural and acceptable way to elicit autonomic arousal and implement IE. Intense exercise produces physiological responses similar to those observed in anxious states, such as increased heart and respiratory rates, but these sensations are typically experienced within a context associated with health benefits rather than danger (24, 25). Despite this conceptual overlap, our review of the literature identified only one study that examined exercise in PD, in which a 30-minute treadmill task was used before *in vivo* exposure sessions as part of a standardized 7-week CBT program for PD with agoraphobia (26). In that trial, exercise functioned as an adjunct component rather than as the primary IE strategy, and no PD-specific outcome scale was employed.

To date, a standardized protocol for using intense exercise as IE in the treatment of PD has not been established, and the direct use of physical exercise as an IE intervention has not been systematically evaluated. Therefore, the present study aimed to examine the feasibility and efficacy of a brief intermittent intense exercise (BIE) program—characterized by repeated bursts of intense activity—as a stand-alone interoceptive exposure strategy for patients with PD. Jacobson’s progressive muscular relaxation training (RT) (27) was chosen as a credible comparison condition matched for time and therapist contact, but without structured exposure to feared bodily sensations.

## Materials and methods

### Patient and public involvement

All study participants were informed of the objectives, methods, potential risks and benefits of the study and provided written informed consent. The Department of Psychiatry of the University of São Paulo Medical School and the Hospital Ethics Committee (CAPPesq protocol n. 742/05) approved the study protocol. The study received a research grant towards its total costs by the São Paulo State Foundation for the Development of Science (FAPESP, project n. 2008/06311-0).

### Study design

This was a prospective, parallel-group, randomized, assessor-blinded clinical trial with two arms (BIE vs RT), conducted at the Anxiety Disorders Program of the University of São Paulo Medical School, Sao Paulo-SP, Brazil.

The trial was registered at ClinicalTrials.gov (identifier NCT06073691).

It compares the effectiveness of two interventions: interoceptive exposure, provided by a protocol of BIE, performed as regular and controlled systematic physical exercise to provide IE compared to a credible control treatment, the RT (27).

All patients received identical placebo pharmacological treatment. Patients and raters were informed that the participants might receive an active pharmacological treatment or a matching placebo. However, all patients received only the placebo pill for up to six months.

The study was conducted at the Anxiety Disorders Program of the Institute of Psychiatry and at the Institute of Orthopaedics and Traumatology of the University of Sao Paulo Medical School, Sao Paulo, Brazil.

### Diagnostic ascertainment and differential diagnosis

All participants completed the full Mini-International Neuropsychiatric Interview (MINI) administered by trained staff, followed by a comprehensive clinical evaluation conducted by a board-certified psychiatrist with expertise in anxiety disorders. The psychiatrist confirmed the DSM-IV-TR diagnosis of panic disorder (with or without agoraphobia), reviewed differential diagnoses, and excluded primary psychotic disorders, bipolar spectrum disorders, current substance-induced conditions, and clinically significant medical or neurological causes that could account for panic-like symptoms. Medical screening included the PAR-Q and a symptom-limited treadmill test under cardiology supervision to identify potential cardiovascular contraindications to the exercise protocol. An experienced psychiatrist, blinded to treatment allocation, conducted all outcome assessments throughout the trial.

### Subjects

The subjects were referred for treatment to the Anxiety Disorders Program from the emergency room of the Cardiology Institute of the University of São Paulo Medical School. After initial clinical interviews of 121 suitable individuals, 102 were found to meet the following inclusion criteria: (1) diagnosis of PD with or without agoraphobia, based on the Mini International Neuropsychiatric Interview (MINI) (28) in accordance with the DSM-IV-TR (29) criteria, and (2) no current medical or any other treatments for PD for the last 12 weeks. The exclusion criteria were (1) clinically relevant risk of cardiovascular disease (according to the Physical Activity Readiness Questionnaire - PAR-Q scale) (30), (2) practice of regular physical exercise for ≥ 150 minutes per week; (3) history or current substance abuse or dependence; (4) pregnancy; (5) breastfeeding; and (6) clinically relevant suicidal ideation or previous suicide attempts. Upon inclusion, participants were assigned to sequential numbers which were previously randomly allocated to either BIE or RT with a Microsoft Excel spreadsheet. The final allocation was BIE (n = 51) and RT (n = 51). Seventy-two patients completed the trial, 38 females (52.8%) and 34 males (47.2%), aged between 21 and 51 years (mean ± SD: 33.3 ± 7.7 years). Among them, 22 (30.6%) were smokers. Regarding level of education and marital status, 47 (65.3%) had college or higher education and 44 (61.1%) were married. The mean age of onset of the panic attacks was 28.3 ± 4.5 years. There were no differences between groups in sociodemographic or clinical characteristics (**Table 1**).

**Table 1:** Clinical and socio-demographic characteristics of completers in the two groups at baseline.

Characteristics		GROUP						X2/U	p-value
		BIE		RT		Total			
		N/Mean	%/SD	N/Mean	%/SD	N/Mean	%/SD		
Sex	Female	19	51.4%	19	54.3%	38	52.8%	0.06	0.803
	Male	18	48.6%	16	45.7%	34	47.2%		
Smoking	No	27	73.0%	23	65.7%	50	69.4%	0.45	0.504
	Yes	10	27.0%	12	34.3%	22	30.6%		
Level of Schooling	< University	12	32.4%	13	37.1%	25	34.7%	0.18	0.675
	> University	25	67.6%	22	62.9%	47	65.3%		
Occupational Status	Not Working	13	35.1%	19	54.3%	32	44.4%	2.67	0.102
	Working	24	64.9%	16	45.7%	40	55.6%		
Marital Status	Not Married	13	35.1%	15	42.9%	28	38.9%	0.45	0.502
Married		24	64.9%	20	57.1%	44	61.1%		
Age		33.5	8.4	32.9	7.1	33.3	7.7	637	0.910
Age of disorder onset		28.8	4.3	27.8	4.6	28.3	4.5	593.5	0.544
Panic Agoraphobia Scale (PAS)		30.5	8.1	27.8	9.2	29.2	8.7	0.88	0.382
Frequency of PA		2.7	1	2.5	1.1	2.6	1.1	1.54	0.129
Intensity of PA		3.7	1.9	3.4	1.7	3.6	1.8	0.91	0.368

P-values obtained with Chi-squared test for categorical variables and Mann–Whitney U test for continuous variables. The data are presented as N and % for categorical variables, and as mean and standard deviation for continuous variables.

PA = Panic Attacks

BIE = Brief intermittent intense exercise group.

RT = Relaxation Training group.

SD: Standard deviation.

**Figure 1:**
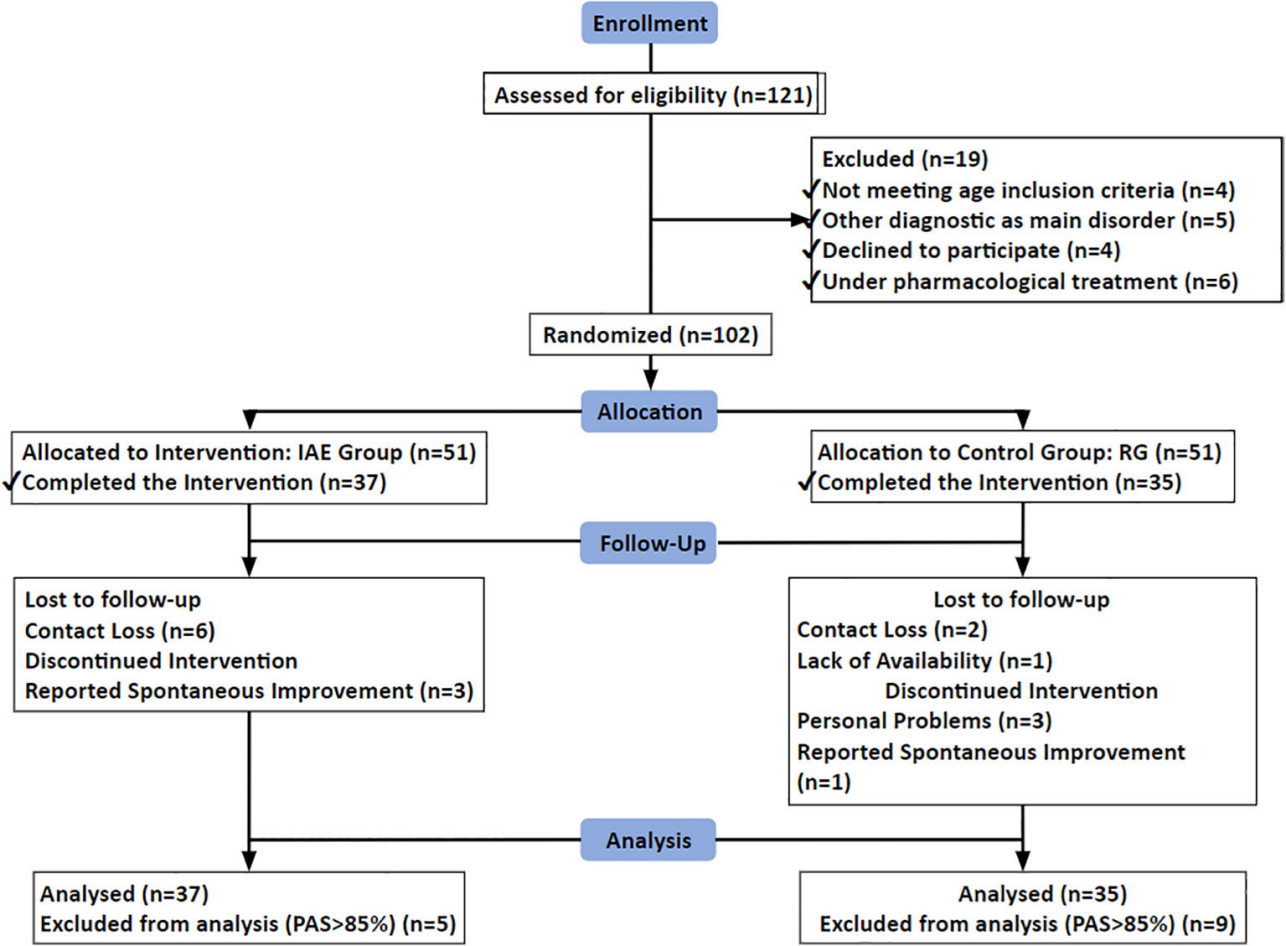
Flowchart of study participants.

The CONSORT flowchart for patient allocation and the reasons for exclusion are shown in **Figure 1**.

### Cardiovascular status assessments

All subjects were assessed for cardiovascular risk with the Physical Activity Readiness Questionnaire (PAR-Q) (30). All subjects then performed a stress test on a medical-grade treadmill (Inbramed, ATL – 10200) at various speeds and inclinations using the Heck’s protocol due to its minimal risk of cardiac events (31).

### Outcome assessments

The study rater was a trained psychiatrist with previous experience using the MINI questionnaire and the symptom severity rating scales used in this project. The rater was blinded to the patient’s allocation. The same rater did all the ratings. All patients were evaluated at presentation (week -2), baseline (week 0), and then at weeks 6, 12, and 24 (follow-up assessment, FU). The intervention programs were performed weekly from week 0 to week 12.

The primary outcome measure was PD severity scores as assessed using Bandelow’s Panic Agoraphobia Scale (PAS) (32, 33). The PAS is a 13-item measure of PD symptoms severity. The observer-rated version was used in the present study. The items assessed by the PAS are PA, agoraphobic avoidance, anticipatory anxiety, disability, functional impairment, and health concerns.

Secondary outcome measures were (1) Frequency and intensity of PA, measured using a PA log (34); (2) Severity of the general anxiety symptoms, assessed with the Hamilton Anxiety Rating Scale (HAM-A) (35, 36), (3) Severity of depressive symptoms, assessed with the Hamilton Depression Rating Scale (HAM-D) (37).

### Treatment protocol

Experimental group (BIE): the training sessions commenced with a 5-minute warm-up and stretching routine, followed by a 15-minute moderate-paced walk, a brief 30-second high-intensity jog, and concluded with another 15-minute walk. The frequency of short sprints increased every two weeks from one to six by the end of the training period. These sprints were alternated with 4.5-minute walking intervals during the 30-minute session. Participants’ cardiovascular capacity determined the exercise intensity, monitored using a Polar RS300X device for accuracy and safety.

Control group (RT): We used the Jacobson Progressive Muscular Relaxation Training (27), conducted by an experienced psychologist from the Anxiety Disorders Program. Three 45-minute sessions were held each week for a duration of 12 weeks. The RT consists in a first step of deep breathing for 3 to 5 times followed by a second step that involves systematically tensing and then relaxing 9 different muscle groups in the body (hands, arms, shoulders, neck, face, chest, abdominal, legs and feet) while focusing on the sensation of relaxation (27).

### Data analysis

The data were described using measures of central tendency and dispersion for continuous variables (mean and standard deviation) and absolute and relative frequencies for categorical variables. The association between categorical variables was assessed using Pearson’s chi-square test.

Comparisons between different groups regarding continuous variables were made using statistical tests such as Student’s t-test for independent samples (when normality was assumed) or the Mann-Whitney U test (for non-normal distributions). A Two-Way Repeated-Measures ANOVA was employed to evaluate differences within and between groups over time. To account for multiple comparisons, a post-hoc analysis with Holm’s correction was applied to control for type I error. The sample size required for the study was determined to be 36 participants per group to achieve a statistical power of 95%, factoring in a moderate effect size(f = 0.25) and an anticipated dropout rate of up to 10%, This calculation was conducted using G*Power software, version 3.1.9.6 (University of Düsseldorf). The statistical analyses were carried out using Jamovi software, version 2.2.5, with a significance level set at 5% (p < 0.05).

## Results

### Primary efficacy measure: PD severity scores

#### Participant flow and baseline characteristics

Of the 121 patients initially screened, 102 met inclusion and exclusion criteria and were randomized to BIE (n = 51) or RT (n = 51). Seventy-two participants completed the 12-week intervention and the 24-week follow-up assessments (BIE: n = 37; RT: n = 35). Only three of the 72 enrolled subjects (4.2%) did not complete the trial. The CONSORT flowchart detailing recruitment, allocation, follow-up, and analysis is presented in **Figure 1**.

At baseline, there were no significant differences between the BIE and RT groups in sociodemographic or clinical characteristics, including age, sex, marital and occupational status, education level, smoking status, age of disorder onset, PD severity (PAS), frequency or intensity of panic attacks (all p > 0.10; **Table 1**).

##### Primary outcome: panic disorder severity

Repeated-measures ANOVA on PAS scores showed a robust main effect of time (F = 207.1, p < 0.001, η² = 0.76), indicating a decrease in PD severity across assessments, and a significant main effect of group (F = 5.1, p < 0.001, η² = 0.07). Importantly, there was a significant group × time interaction (F = 56.1, p < 0.001, η² = 0.46), indicating differential trajectories of improvement in the two treatment arms.

**Figure 2:**
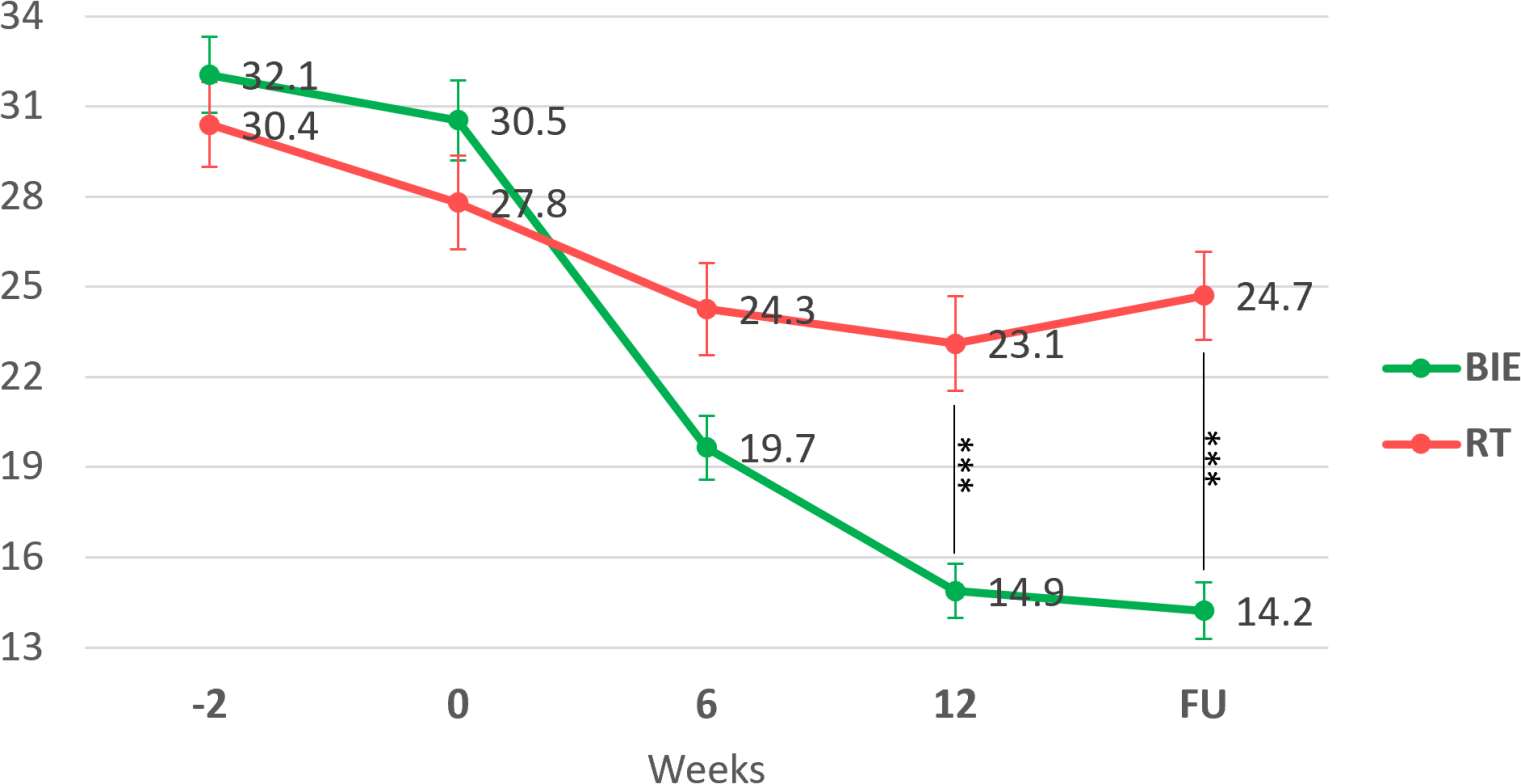
Panic Agoraphobia Scale (PAS) changes scores in the intervention (BIE) and control (RT) groups. Dots and whiskers indicate mean and standard error, respectively.***p < 0.001 pairwise between group significance level. BIE, Brief Intermittent Intense Exercise group; RT, Relaxation Training group; FU, follow-up evaluation (week 24).

Both groups showed reductions in PAS scores from pre-intervention (weeks −2 and 0) to post-intervention (week 6, endpoint at week 12) and follow-up (week 24). However, *post hoc* comparisons revealed that the BIE group had significantly lower PAS scores than the RT group at week 12 (BIE: 14.9 ± 5.3 vs. RT: 23.1 ± 9.4; t = −4.72, p < 0.001) and at follow-up (BIE: 14.2 ± 5.5 vs. RT: 24.7 ± 8.5; t = −6.07, p < 0.001) (**Figure 2**).

To account for the small number of dropouts, an intention-to-treat analysis was conducted using the last observation carried forward for missing data; this analysis yielded a similar pattern of results.

##### Secondary outcomes: frequency and intensity of panic attacks

Both the frequency and intensity of panic attacks decreased from pre-intervention to week 12 and then increased again from week 12 to the 24-week follow-up in both groups. However, from week 12 to follow-up, the increase in frequency and intensity of panic attacks was more pronounced in the RT group.

**Figure 3:**
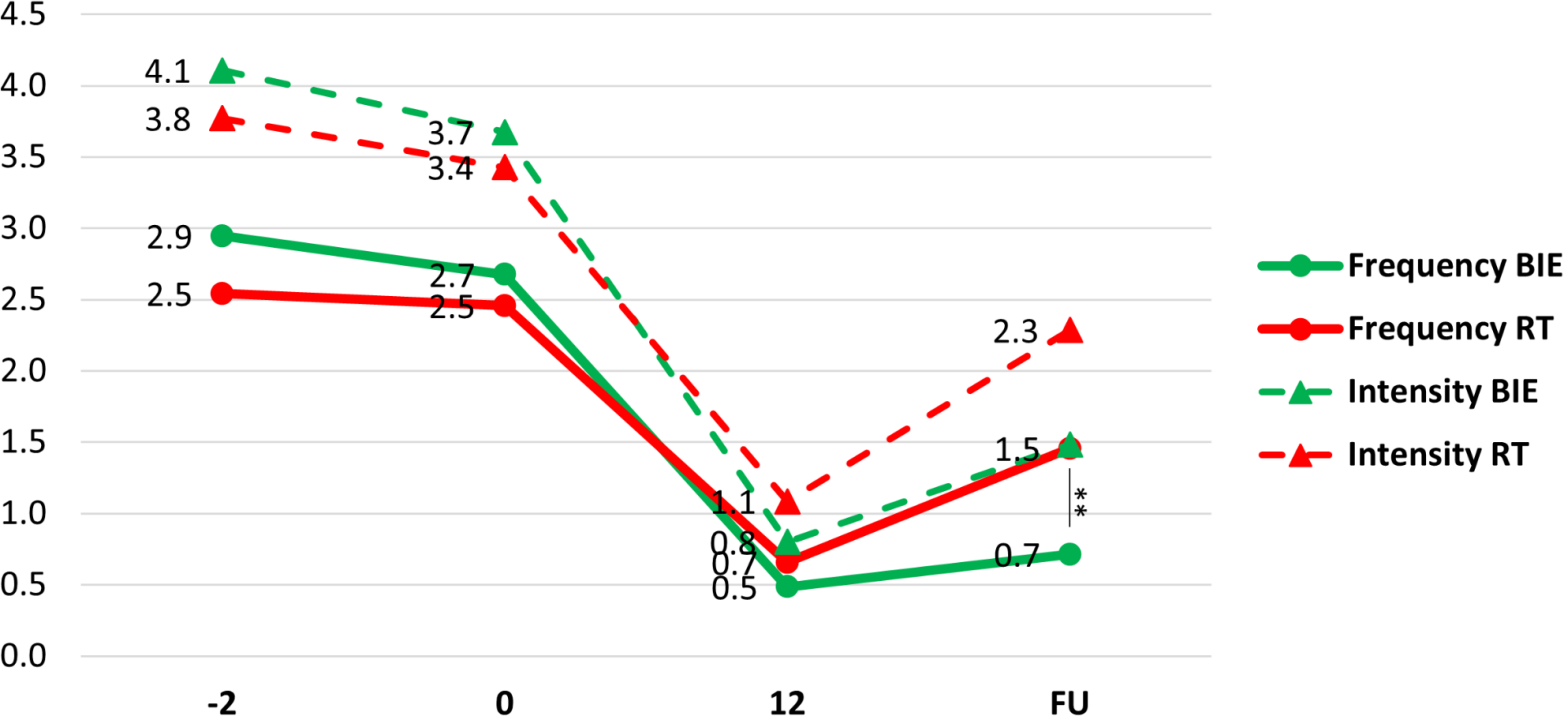
Frequency and intensity of panic attacks (PA) changes in groups. Means of panic attack frequency (circles) and intensity (triangles) in groups.**p = 0.003 pairwise between group significance level. BIE, Brief Intermittent Intense Exercise group; RT, Relaxation Training group; FU, Follow-up evaluation (week 24).

There was a significant group × time interaction for both frequency (F = 7.96, p < 0.001, η² = 0.105) and intensity (F = 3.336, p = 0.043, η² = 0.047) of panic attacks (**Figure 3**). At follow-up, the BIE group experienced fewer panic attacks than the RT group (BIE: 0.7 ± 0.6 vs. RT: 1.5 ± 1.0; t = 3.79, p = 0.003).

##### Secondary outcomes: general anxiety and depressive symptoms

For general anxiety and depressive symptoms, repeated-measures ANOVA showed significant group × time interaction effects for both the Hamilton Anxiety Rating Scale (HAM-A: F = 5.041, p = 0.005, η² = 0.069) and the Hamilton Depression Rating Scale (HAM-D: F = 13.967, p < 0.001, η² = 0.170).

**Figure 4:**
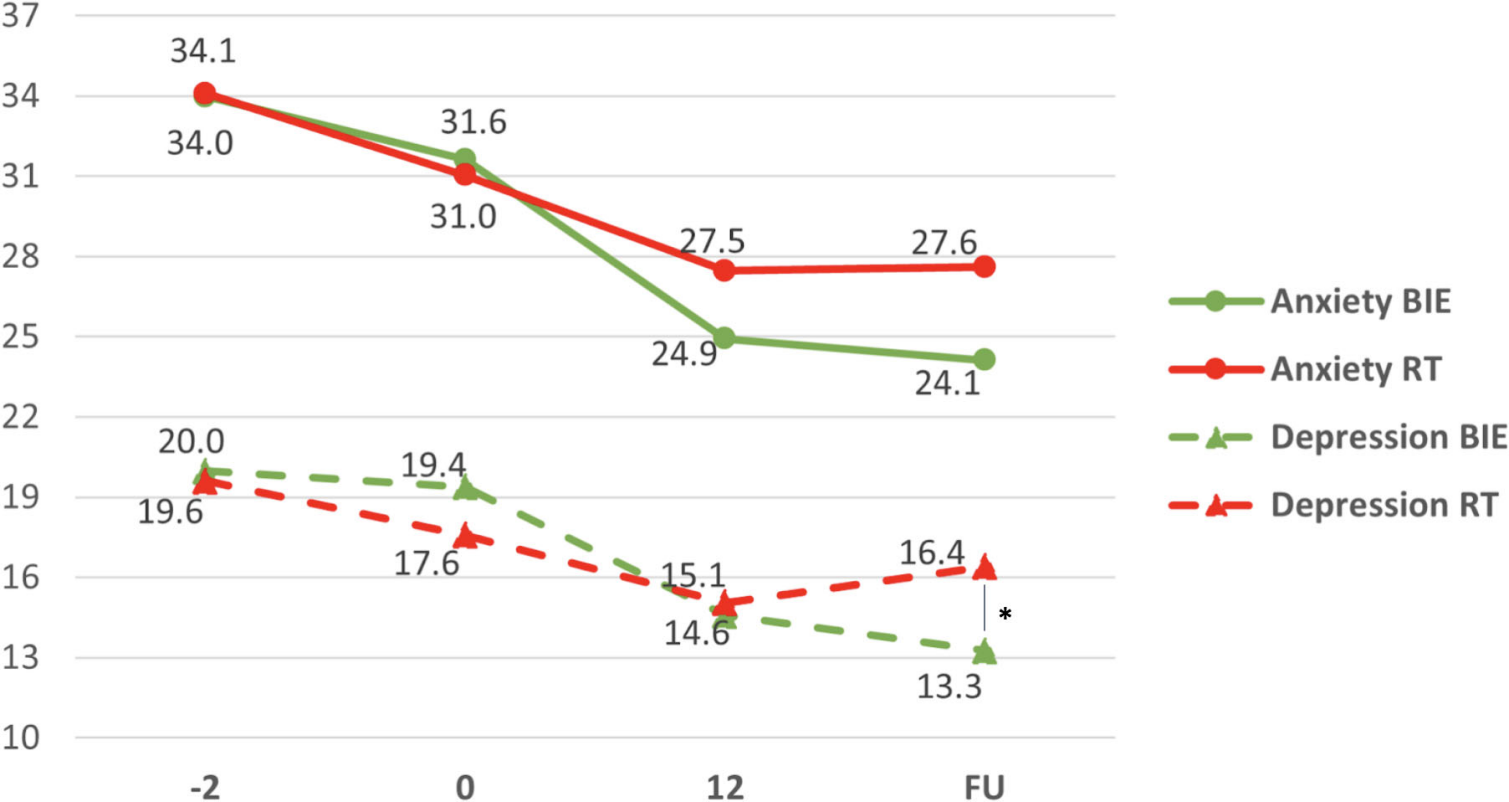
General anxiety and depressive symptoms change scores in intervention (BIE) and control (RT) groups. Mean of anxiety (circles) and depression symptoms (triangles) in groups. *p = 0.013 pairwise between group significance level. BIE, Brief Intermittent Intense Exercise group; RT, Relaxation Training group; FU, Follow-up evaluation (week 24).

There were also strong main effects of time, indicating overall symptom reduction across the trial (HAM-A: F = 85.447, p < 0.001, η² = 0.557; HAM-D: F = 78.869, p < 0.001, η² = 0.537). Between-group differences were most evident at follow-up for depressive symptoms: at week 24, HAM-D scores were lower in the BIE group compared with the RT group (13.3 ± 4.7 vs. 16.4 ± 5.6; t = −2.552, p = 0.013) (**Figure 4**).

## Discussion

This randomized clinical trial examined the efficacy of interoceptive exposure (IE) delivered through a brief intermittent intense exercise (BIE) protocol in comparison with Jacobson’s relaxation training (RT) in treatment-free patients with panic disorder (PD). Overall, both interventions led to symptomatic improvement; however, BIE was associated with greater and more sustained reductions in PD severity and panic attack frequency, as well as more favorable depressive symptom outcomes at 6-month follow-up.

### Primary outcome: panic disorder severity

Both groups showed a decrease in PD severity over time, as reflected in PAS scores, but the trajectories differed. At week 12, the BIE program yielded significantly greater symptom reduction than RT, and this between-group difference persisted at the 24-week follow-up. Patients in the BIE group also experienced fewer panic attacks at follow-up, suggesting that the benefits of exercise-based IE extend beyond global severity ratings.

These findings reinforce the notion that IE is a core component of effective PD treatment protocols (16). Nonetheless, there is substantial variability in how IE is implemented in clinical practice, and some clinicians adopt a cautious stance due to concerns about potential adverse effects (38). In the present trial, IE was delivered in an intensive, systematic manner through BIE, which aligns with Deacon’s observation that more intensive IE can maximize clinical gains, particularly in reducing respiratory and overall anxiety indices (38).

Consistent with this, a component network meta-analysis by Pompoli et al. (39) across 72 CBT trials for PD found that treatment combinations incorporating IE were associated with higher odds of short-term remission, whereas relaxation components were linked to lower efficacy. Our results extend this literature by demonstrating that, when directly compared in a controlled design, an exercise-based IE protocol was more effective than RT on a PD-specific outcome (PAS).

### Frequency and intensity of panic attacks

The frequency and intensity of panic attacks decreased in both groups from pre-intervention to week 12 and then increased again from week 12 to follow-up. Importantly, this rebound was more pronounced in the RT group. This pattern is compatible with previous evidence that the benefits of RT on anxiety symptoms tend to attenuate over time (39), whereas exposure-based approaches may yield more durable changes in threat learning and symptom appraisal.

### Anxiety and depressive symptoms

General anxiety (HAM-A) and depressive symptoms (HAM-D) decreased over time in both groups, with significant group × time interactions. The between-group difference was most evident for depressive symptoms at follow-up, when the BIE group showed lower HAM-D scores than the RT group. While both interventions may confer non-specific benefits on mood, the continued improvement in the BIE group after week 12, contrasted with the relative worsening in the RT group, again suggests more sustained gains with exercise-based IE.

### Persistence of therapeutic effects

A clinically relevant finding is the maintenance of BIE benefits up to six months after treatment initiation (week 24). One plausible explanation is that repeated pairing of intense physiological arousal with a safe and controllable context promotes new learning about bodily sensations as non-dangerous (24, 25). This reinterpretation of interoceptive cues may persist beyond the structured intervention and generalize to daily-life situations in which somatic arousal is experienced.

Previous work from this group using office-based IE also reported long-lasting treatment effects, with benefits maintained for up to one year (19), and Keough and Schmidt (20) observed largely sustained reductions in anxiety sensitivity six months after a brief IE-based intervention. The present study adds to this evidence by showing similar durability of effects when IE is embedded in a structured exercise program.

### Treatment adherence

Treatment adherence in this study was notably high compared with prior PD trials (40, 41). Only three participants discontinued the protocol. One possible explanation is that patients found the acquisition of health-related behaviors and self-management strategies intrinsically rewarding (42). Additionally, contextual factors may have enhanced adherence; participants were recruited at the emergency room of the Cardiology Institute and then treated at the Psychiatric Institute, with part of the intervention conducted at the Movement Laboratory of the Orthopaedics Institute, a setting typically associated with elite sports evaluations. Such a high-status, health-oriented environment may have contributed to motivation and retention.

### Use of relaxation training as a control condition

RT is an established intervention for anxiety symptoms, particularly in generalized anxiety disorder. However, it is not considered a first-line or highly effective treatment for PD, and its benefits tend to decrease over time (39, 43, 44). For this reason, RT has been adopted as a control condition in several PD trials (39, 43, 44). In the present study, RT served as a credible psychological placebo with strong face validity for patients, while allowing a conservative comparison with an active, theoretically grounded IE intervention. The finding that BIE outperformed RT despite this credibility advantage underscores the specific value of exercise-based IE for PD.

### Metabolic effects of exercise and panic threshold

Interestingly, sedentary PD patients in this sample did not experience panic attacks during intense exercise, despite evidence of abnormal responses to the Heck treadmill protocol (31). From a theoretical standpoint, one might expect panic attacks during intense exercise due to hyperventilation-induced changes in respiratory frequency and the suffocation false alarm model of PD (45). However, most hyperventilation-induced panic episodes have been reported in laboratory or office-based procedures (46, 47).

In this trial, it is possible that respiratory alkalosis associated with hyperventilation was at least partially offset by exercise-induced metabolic acidosis, attenuating the panicogenic effect of pH shifts. Alternatively, the highly controlled and medically supervised laboratory environment may have provided strong safety cues, thereby reducing the likelihood of panic despite intense physiological arousal.

### BIE as an interoceptive exposure strategy in PD

To our knowledge, no previous study has evaluated BIE as a stand-alone IE strategy for PD. Preliminary evidence suggested that acute bouts of exercise may have anti-panic effects in both healthy individuals (48, 49) and patients with PD (25, 50). However, direct comparisons with the only prior PD exercise study are limited because of differences in design, objectives, and outcome measures, particularly the absence of a PD-specific rating scale in that trial (26). Nevertheless, that study also reported a group × time interaction on non-specific anxiety symptoms (HAM-A), with greater improvements in the more intense exercise condition, converging with our findings.

### Study limitations

This study has several limitations. First, in order to maximize effect sizes, we specifically recruited sedentary PD patients who were also naïve to exercise stress tests. The generalizability of our findings to patients who already exercise regularly or are less fearful of somatic arousal is therefore limited. Individual responses to IE are likely to vary, and the intervention may be particularly beneficial for those with high fear of bodily sensations and their perceived consequences (51). Future trials should include more heterogeneous samples to clarify moderators of treatment response.

Second, we used pill placebo in both groups. This is uncommon in non-pharmacological randomized controlled trials, especially when the main comparison involves psychological or behavioral interventions. The relative efficacy of pharmacological versus non-pharmacological treatments for PD is already well established (52), and our intent was to approximate the clinical experience of patients who typically receive combined pharmacological and psychosocial care. However, resource constraints prevented the implementation of a four-arm design that could have fully disentangled pill-placebo and psychological-placebo effects. Additional studies are needed to examine the potential additive or interactive effects of BIE in patients receiving active pharmacotherapy.

Third, the follow-up period was limited to six months (12 weeks of treatment plus 12 weeks of follow-up). Given that anxiety disorders often follow a chronic, recurrent course (53), longer-term studies are required to determine whether the advantages of BIE over RT are maintained over years and how best to support continued exercise adherence.

Finally, although diagnoses were established with the full MINI and confirmed by a board-certified psychiatrist, we did not obtain an independent second clinical verification. Future trials could strengthen diagnostic rigor by incorporating consensus conferences or multi-informant assessments. Inclusion criteria was panic disorder with or without agoraphobia. As a limitation to the present study findings, we cannot rule out the possibility that patients with panic disorder, with or without agoraphobia, could have different outcomes.

### Future directions and scalability

Exercise can be prescribed and supervised by various categories of health professionals with relatively modest additional training, which enhances the scalability of BIE-type interventions. At the same time, other delivery formats—such as virtual reality-based exposure (54, 55) and internet-delivered programs (56)—offer promising avenues for disseminating IE-informed treatments and may be combined with exercise-based protocols in stepped-care models. Further research is needed to evaluate these hybrid approaches and to identify optimal ways to integrate BIE into routine care for PD.

## Conclusion

In this randomized, assessor-blinded clinical trial, a 12-week brief intermittent intense exercise (BIE) program, used as an interoceptive exposure strategy, was more effective than Jacobson’s relaxation training in reducing panic disorder severity, decreasing the frequency of panic attacks, and improving depressive symptoms, with benefits maintained up to 24 weeks. These findings support the clinical relevance of delivering interoceptive exposure through a structured, supervised exercise protocol for treatment-free patients with panic disorder.

Because BIE can be implemented in outpatient settings with relatively simple resources and supervised by different categories of health professionals, it represents a potentially scalable, low-cost, and health-promoting addition to existing treatment options for panic disorder. Future studies should examine long-term maintenance of gains, identify patient characteristics associated with better response, and evaluate the integration of exercise-based interoceptive exposure into stepped-care and combined pharmacological–psychological treatment models.

## Data Availability

The original contributions presented in the study are included in the article/**Supplementary Material**. Further inquiries can be directed to the corresponding author.
